# Engineering the effector specificity of the macrolide-sensing transcription factor MphR for the detection of sesquiterpenes

**DOI:** 10.64898/2026.10.16.759199

**Published:** 2026-10-19

**Authors:** Cassidy L. Oliverio, Alissa M. Meo, Stefanie Chen, Gavin J. Williams

## Abstract

Terpenes are a large, diverse class of natural products used as flavors, fragrances, pharmaceuticals, and more, yet obtaining them often requires either large quantities of biomass or complex chemical reactions. Directed evolution of key enzymes and pathways could improve biosynthetic yields, but most sesquiterpenes lack high-throughput screening methods to search large libraries of variants. To alleviate this bottleneck, we developed the first reported genetically encoded biosensor to detect four highly valuable sesquiterpenes: valencene, nootkatone, artemisinin, and cedrol. Starting with the macrolide transcription factor biosensor MphR, we screened a panel of mutants against the four sesquiterpenes, including MphR-13 (T17N/V18I/K21L/S106L/H147L/P161L), which readily detected all four sesquiterpenes. Computational modeling showed that the mutations reduced the size of the ligand-binding pocket compared with the wild type, which positively correlated with the biosensor’s fluorescence output. Additionally, swapping the fluorescence reporter improved the sensor’s response 15-20-fold, yielding a generalist sesquiterpene biosensor that can be further developed and applied to the directed evolution of sesquiterpene biosynthesis.

